# Acute radiodermatitis in modern adjuvant 3D conformal radiotherapy for breast cancer - the impact of dose distribution and patient related factors

**DOI:** 10.1186/s13014-018-1160-5

**Published:** 2018-11-07

**Authors:** Kai J. Borm, Maximilian Loos, Markus Oechsner, Michael C. Mayinger, Daniela Paepke, Marion B. Kiechle, Stephanie E. Combs, Marciana N. Duma

**Affiliations:** 1 Department of Radiotherapy, Klinikum Rechts der Isar, Technical University, Munich, Germany; 2 Faculty of Medicine, Technical University, Munich, Germany; 3 Department of Gynecology and Obstetrics, Klinikum Rechts der Isar, Technical University, Munich, Germany; 4 Deutsches Konsortium für Translationale Krebsforschung (DKTK)-Partner Site Munich, 81675 Munich, Germany; 5 Institute of Innovative Radiohterapy, Helmholtzzentrum München, Munich, Germany; 6 Department of Radiation Oncology, Klinikum rechts der Isar/ TU Munchen, Ismaninger Strasse 22, 81675, Munchen, Germany

**Keywords:** Breast cancer, Radiodermatitis, 3D-CRT, Prognostic factors, Skin

## Abstract

**Purpose:**

This study was performed to evaluate skin toxicity during modern three-dimensional conformal radiotherapy (3D-CRT) and to evaluate the importance of dose distribution and patient related factors.

**Material and methods:**

This study comprises 255 patients with breast cancer treated with tangential three-dimensional conformal radiotherapy (3D-CRT) after breast conserving surgery between 03/2012 and 05/2017. The median prescribed dose was 50.4 Gy (range 50–50.4) and 92.2% of the patients received a sequential boost of 10–16 Gy. Adverse skin toxicities (according to CTCAE v. 4.03 and the occurrence of moist desquamations) were assessed at the end of treatment. The dose distribution in the skin (5 mm strip from the patient outline) and in the CTV was evaluated and correlated to the CTCAE scores and the occurrence of moist desquamation.

**Results:**

42.4% of the patients developed grade I, 55.7% grade II and 2% grade III skin toxicities. Moist desquamation was observed in 59 cases (23.1%). Dose distribution within the CTV and skin was homogenous with only small areas receiving 107% of the prescribed dose (median: 0.7 cm^3^) in the CTV and 105% (median 0.5 cm^3^) in the skin. On univariate analysis breast size as well as V107%(CTV), V105%(skin) and V80%(skin) correlated significantly (*p* < 0.05) with the incidence of skin toxicity. On multivariate analysis only V80%(skin) was confirmed as independent risk factor.

**Conclusion:**

Modern tangential multi-field 3D-CRT allows a homogeneous dose distribution with similar skin toxicity as compared to studies performing IMRT. Dose distribution within the skin (V80%) might have a relevant impact on the severity of skin toxicity and the occurrence of moist desquamation.

## Introduction

Adjuvant radiotherapy is an indispensable part in the therapy of early breast cancer. Meta-analyzes and randomized trails show a significant improvement in regard to local control rates and breast cancer specific survival [1]. However 74–100% of the patients undergoing radiotherapy of the breast develop skin irritations such as erythema, desquamation and edema [2, 3]. Related complaints such as pain, itching and burning are shown to lower the quality of live during and after the radiotherapy treatment [4]. Furthermore several studies indicate that acute skin reactions are a relevant risk factor for the occurrence of late skin toxicities [5, 6]. Skin toxicity affects almost all patients who undergo adjuvant radiotherapy in breast cancer. However, the degree of severity varies largly [7, 8].

Previous studies identified patient-related factors (smoking, breast size and body mass index) as well as factors related to the treatment procedure (e.g. concomitant hormone treatment and dose distribution) to be predictive of severe side effects [8–10]. According to Chen et al. [11] and Tortorelli et al. [12] dose inhomogeneities of > 107% and > 110% within the target volume are one of the most important predictors for acute skin damage. In accordance with this, radiotherapy techniques that enable a more homogenous dose distribution such as intensity modulated radiation therapy (IMRT) are associated with lower side effects [13, 14]. Therefore many clinics try to keep the high-dose areas as low as possible especially when 3DCRT treatment planning is used. Pastore et al. [15] showed that also the dose distribution within the skin during 3DCRT has an impact on the occurrence of skin toxicities. However, the existing data on dose distribution, patient related factors and the occurrence of skin toxicity is sparse. This study was performed to evaluate skin toxicity with modern 3D-CRT in a large patient collective with special emphasis on the dose distribution within the skin and the target volume.

## Methods and material

### Patient population

Two hundred fifty-five patients with breast cancer treated in our institute between 03/2012 and 05/2017 were included in this study. All patients underwent breast-conserving therapy prior to radiotherapy. In 102 (40.0%) cases, chemotherapy was part of the treatment concept, with 73 (28.6%) patients treated after (adjuvant) and 29 (11.4%) patients before surgery (neoadjuvant). The median age of the patient collective was 55.0 (23–85) years. 131 (51.4%) tumors were located in the right breast, 122 (47.8%) in the left breast. Twenty-four of 255 patients (9.4%) were smokers (daily or at least regular consumption). The patients’ characteristics are summarized in Table 1.

**Table 1:** Patients’s characteristic table

Variable	
Age in years (median and range)	55.0 (23–85)
Side of breast cancer	
Right	131 (51.6%)
Left	121 (47.6%)
Smoker	
Yes	24
No	231
Chemotherapie	102 (40%)
Neoadjuvant	29 (11.4%)
Adjuvant	73 (28.6%)
Time in days between Chemo- and Radiotherapy	70 (19–252)
Breast size in cm^3^ (median and range)	568 (100.0–2350.6)
CTCAE	
I°	108 (42.4%)
II°	142 (55.9%)
III°	5 (2.0%)
Moist Desquamation	
Yes	59 (23.2%)
No	195 (76.8%)
Boost	
Yes	235 (92.2%)
No	20 (7.9%)

### Treatment planning

All patients underwent a planning kilo-voltage computed tomography (kVCT) scan (Siemens medical solutions, Erlangen, Germany) in free breathing with an axial slice thickness of 3 mm. The contouring and treatment planning was performed with the Eclipse Treatment Planning System Version 10.0 (until 04/2015) or 13.0 (Varian Medical Systems, Palo Alto, CA, USA). For each patient the mammary gland was contoured and defined as CTV according to the RTOG guidelines (https://www.rtog.org/CoreLab/ContouringAtlases/BreastCancerAtlas.aspx). Additionally all organs at risk such as the lung and the heart were contoured. The patients were treated using 3D-CRT with an individually optimized plan for each patient. All plans consisted of 2 opposing tangential beams. If necessary, wedges were applied. Additional beam segments (1–7) were used to improve target dose coverage and homogeneity. Dose calculation was performed using the anisotropic analytical algorithm (AAA) Version 10.028 with heterogeneity correction. The prescribed dose was 50.4 Gy (single dose 1.8 Gy) or 50.0 Gy (single dose 2 Gy). The dose of patients treated with 1.8 Gy per fraction was converted to the 2 Gy dose per fraction equivalent dose by using an α/β of 10 Gy for cutaneous reactions [16]*.* Photon energy was 6 MV in all cases. Two hundred thirty-five patients (92.5%) received a sequential boost of 10 Gy (*n* = 100) or 16 Gy (*n* = 137) to the tumor bed with a CTV to PTV margin of 1 cm. The dose was either prescribed to an ICRU reference point located centrally inside the PTV or to the median PTV dose.

### Studied factors

A “skin” structure was retrospectively contoured in addition to the clinical target volume and the organs at risk. This structure was defined as a 5 mm strip from the patient outline in the area of the chest that received ≥5 Gy. The maximum (Dmax) and mean dose (Dmean) was calculated both for the CTV and for the skin. The absolute volume (cm3) receiving ≥110% (V110%), 107% (V107%) and 105% (V105%) of the prescribed dose to the whole breast was measured for the skin and the CTV. Furthermore we assessed the volume of the skin receiving a minimum of 40 Gy (V80%). The breast size of each patient was estimated on basis of the clinical target volume. Experienced radiooncologists evaluated clinically evident skin toxicities at the end of the radiation therapy using the CTCAE V.4.0 scale (Table 2). Clinical endpoints of our analysis were the degree of radiation dermatitis according to CTCAE and the occurrence of moist desquamation.

**Table 2:** CTCAE criteria for skin toxicity

Common Terminology Criteria for Adverse Events (CTCAE): Adverse effect grade Definition Dermatitis radiation: A finding of cutaneous inflammatory reaction occurring as a result of exposure to biologically effective levels of ionizing radiation.				
I°	II°	III°	IV°	V°
Faint erythema or dry desquamation	Moderate to brisk erythema; patchy moist desquamation, mostly confined to skin folds and creases; moderate edema	Moist desquamation in areas other than skin folds and creases; bleeding induced by minor trauma or abrasion	Life-threatening consequences; skin necrosis or ulceration of full thickness dermis; spontaneous bleeding from involved site; skin graft indicated	Death

### Statistical analysis

Statistical analyses were performed using SPSS Statistics v.23 (IBM Corp, Armonk, USA). Since the number of grade III° skin toxicities was low (*n* = 5) in our collective, grade II° and grade III° were considered as common category for most statistical analyses. The occurrence of CTCAE grade II/III and moist desquamation was correlated to the studied factors mentioned above. Patients were categorized regarding the grade of skin toxicity and the presence of moist desquamation and compared among each other. Univariate and multivariate analyses were performed using a binary logistic regression model. For multivariate regression models we included the significant patient-related co-factors and the significant dose parameters from univariate analysis. The cofactors included in the multivariate regression models were tested for collinearity by calculating the bivariate (Pearson) correlation coefficient.

## Results

**Fig. 1:**
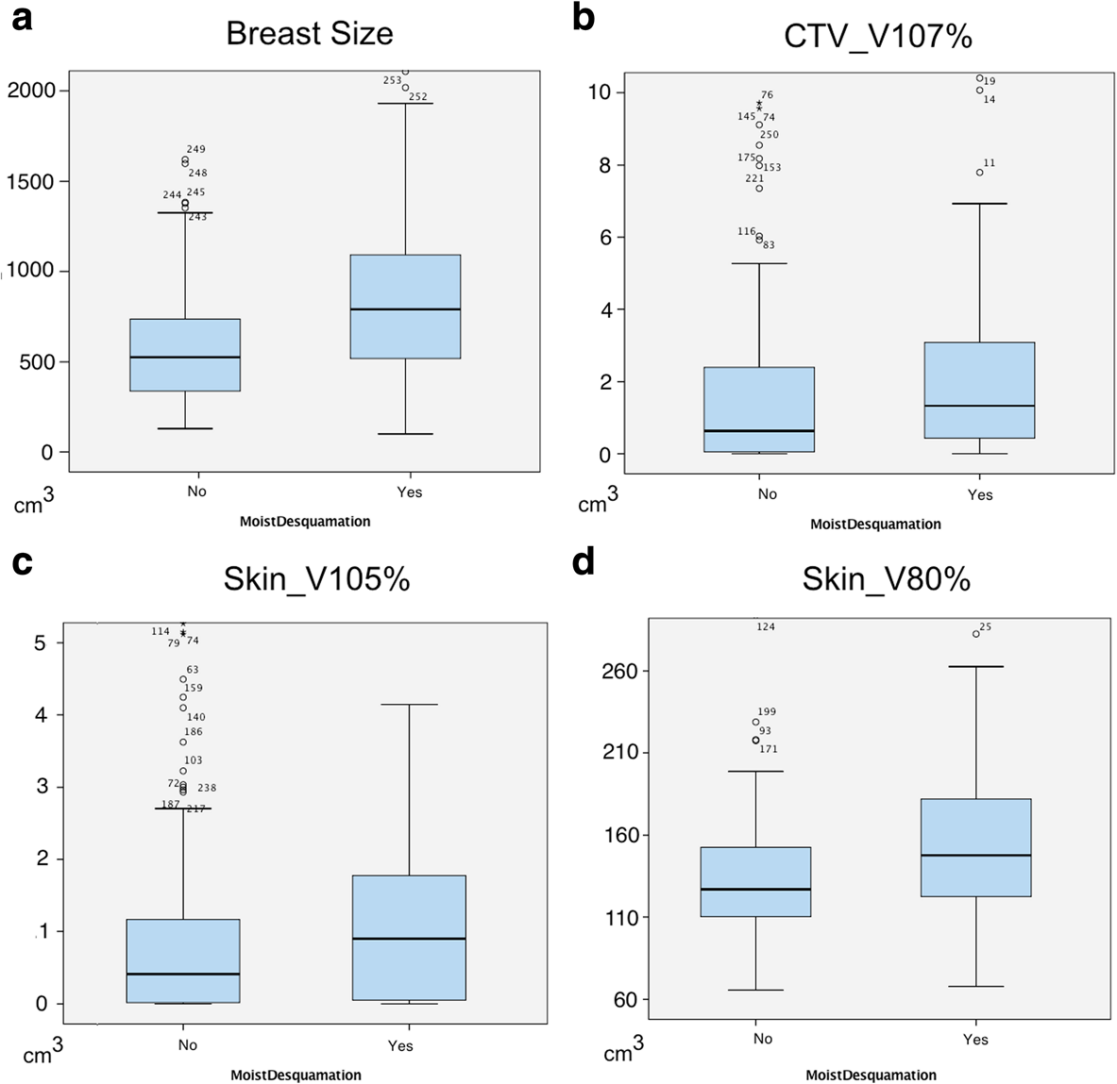
Box Plot Diagrams for all significant factors on the occurrence of moist Desquamation. **a** Breast size; **b** Target volume that receives 107% of the prescribed dose to the whole breast; **c** Skin volume that receives 105% of the prescribed dose to the whole breast. **d** Skin volume that receives 80% of the prescribed dose to the whole breast

All patients showed skin irritations to a certain degree by the end of the treatment. Grade I toxicity was observed in 108 cases (42.4%), grade II toxicity in 142 cases (55.7%) and grade III toxicity in only 5 cases (2%) according to the CTCAE V.4.0 scale. Moist skin desquamation was observed in 59 cases (23.1%). On univariate analyses, a larger breast volume (> median [568 cm3] vs. ≤ median) was significantly associated with the occurrence of acute skin toxicities grade II°-III° or the occurrence of moist desquamation (both *p* < 0.01). Age, chemotherapy prior to treatment, the localization of the tumor (left vs. right), smoking, as well as boost had no significant influence on the development of acute skin toxicities (Table 3). The Dmean in the skin was 36.6 Gy (19.8–38.9 Gy) in the skin. There were only small differences in regard to the maximal dose between CTV and skin (53.0 Gy [47.7–61.8 Gy] vs. 52.9 Gy [48.4–62.0 Gy]). None of assessed dose parameters described above had a significant influence on the severity of skin adverse effects according to CTCAE. However, we observed a significant impact of V107% (CTV); 105% (skin) and V80% (skin) on the occurrence of moist desquamation. The volume of the CTV receiving more than 107% of the prescribed dose was low in this study with an average value of 0.73 Gy (0.0 Gy- 79.0 Gy). Nevertheless, a larger volume receiving a dose of > 107% of the prescribed dose was associated with the occurrence of moist desquamation (*p* = 0.04). For the large majority of the patients (213/255) the maximal dose in the skin was < 107%. Therefore the V105% of the skin was analyzed instead. Similar to the findings in the CTV, a higher V105 of the skin was associated with the occurrence of moist desquamation (*p* = 0.03). Furthermore the skin volume that received 80% of the prescribed dose of 50.0 Gy had a significant impact on the appearance of moist desquamation (*p* = 0.05) and a trend towards a higher appearance of adverse effects CTCAE II°-III° (*p* = 0.06) was observed (Table 4). Figure 1 delineates the dose differences (V107%_CTV; V105%_skin; V80% skin) and the breast volume in regard to the appearance of moist desquamation. Two-parametric multivariate logistic regression models, including breast size and the significant dose parameters, confirmed V80%(skin) as an independent risk factor (HR 1.01 (1.00–1.03), *p* = 0.03; constant − 3.10) for the occurrence of moist desquamation. For V105%_skin (HR 1.08 (0.98–1.19), *p* = 0.10; constant − 1.92) and V107%_CTV (HR 1.01 (0.99–1.03) *p* = 0.29, constant − 2.97) no significant impact was observed. The correlation coefficients between breast size and the dose parameters were *r* = 0.62 (V80%_skin), *r* = 0.14 (V105%_skin) and *r* = 0.04 (V107%_CTV) respectively.

**Table 3:** Univariate analysis to determine factors associated with CTCAE grade II-III toxicity and moist desquamation in 276 patients

Variable	*P* value	
	CTCAE	Moist Desquamation
Age (≤median vs. > median)	0.81	0.25
Neoadjuvant Chemotherapy	0.25	0.37
Adjuvant Chemotherapy	0.94	0.43
Boost	0.16	0.43
Breast size (≤median vs. > median)	0.01^*^	< 0.01^*^
Smoking	0.17	0.48

^*^p < 0.05

**Table 4:** Univariate analysis to assess the impact of dose factors on the appearance of CTCAE grad II-III toxicity and moist desquamation in 276 patients

Variables	CTCAE			Moist Desquamation		
	I°	II°/III°	*P* value	No	Yes	*P* value
Dmax CTV (Gy)	52.9 (50.0–59.8)	52.9 (47.7–61.8)	0.91	52.9 (50.0–61.84)	53.1 (47.7–55.5)	0.34
Dmax skin (Gy)	53.3 (50.2–60.1)	53.4 (48.4–62.1)	0.64	52.4 (47.9–61.0)	52.6 (47.6–54.2)	0.93
Dmean skin (Gy)	36.5 (23.0–38.7)	36.6 (19.8–38.9)	0.66	36.6 (19.8–38.7)	36.4 (20.5–38.9)	0.10
V107% CTV (cm^3^)	0.4 (0.0–79.0)	0.8 (0–45.8)	0.54	0,6 (0–79.0)	1.3 (0–45.8)	0.04^*^
V105% Skin (cm^3^)	0.3 (0.0–8.7)	0.6 (0.0–46.5)	0.27	0.4 (0.0–17.2)	0.9 (0.0–46.5)	0.03^*^
V80% Skin (cm^3^)	124.7 (35.3–293.9)	134.3 (5.9–282.6)	0.06	126.9 (5.9–293.9)	147.7 (67.9–282.6)	< 0.01^*^

Mean values ± SD. ^*^p < 0.05

## Discussion

The prevalence of skin toxicity in the current literature varies widely. This can be attributed to the fact that the definition of radiation dermatitis is inconsistently and different subjective parameters such as erythema, desquamation and edema are used as clinical endpoints. Kraus-Tiefenbacher et al. [7] evaluated the skin toxicity of 211 breast cancer patients treated with 3D-CRT based on the degree of erythema at the end of a 50 Gy course. In 28.9% of the cases no erythema was observed during the treatment, 62.2% showed erythema grade 1 and 8.5% erythema grade 2. Other toxicity criteria such as edema and desquamation were not included in their analysis. Chen et al. [11] on the other hand focused on the incidence of moist desquamation to assess predictive factors of radiation-induced skin toxicity in the 3D-CRT treatment of breast cancer patients. The proportion of patients with radiation-induced moist desquamation was 23% across 158 patients, which is in good accordance with our findings (23.2%). Freedmann et al. [17] used both the “CTCAE score for radiation dermatitis” and moist desquamation as clinical endpoint in their study on IMRT and radiation related skin toxicity. CTCAE grade 1 adverse effects were observed in 30% of the cases, 70% developed grade 2 skin changes. Grade 3 or higher toxicities did not occur. The authors concluded that the CTCAE scoring system is not sensitive to evaluate acute toxicity due to interobserver variability on degree of erythema and that studies should focus on moist desquamation instead. Nevertheless since the CTCAE score is often used in everyday clinical practice we decided to include both CTCAE scores and moist desquamation in this study.

Previous studies identified breast size, body mass index, concurrent hormone therapy and smoking to be predictive factors of radiation-related skin toxicity. This was confirmed in our study in regard to the breast size. The number of patients with a history of smoking was low in our collective, which possibly explains that no significant relationship was found.

The existing data in regard to dose distribution in the target volumen and the skin and the occurance of skin toxicity is sparse. Tortorelli et al. [12] correlated retrospectively in 339 patients’ dose hot spots during 3DCRT with skin toxicities. The authors stated that dose inhomogeneties > 107% within the target volume have a significant impact on the occurrence of severe skin reactions. Chen et al. [11]performed a similar analysis and showed that a V110% of > 5.13% correlates with the incidence of skin toxicity. In accordance with this, treatment planning techniques with a more homogenous dose distribution are shown to result in lower rates of severe skin toxicity: Harsolia et al. [13] showed that the incidence of grade 2 side effects can almost be halved from 80 to 40% by using IMRT (median prescribed dose 45 Gy). Freedman et al. [17] compared in a similar approach a group of 72 breast cancer patients undergoing standard fractionated IMRT with a matched one to one control group of 60 women undergoing conventional photon radiation. They found a significant reduction of moist desquamation (21% vs. 38%) using IMRT.

As hot spots occur often in close proximity to the skin [12], it seems comprehensible that a more homogenous dose distribution results in lower rates of skin toxicity. In “modern” 3DCRT, additional field segments are used to optimize the maximal dose within the target volume. In our collective only 19 patients showed hot spots of > 110% within the target volume and they were all smaller than 5.13% in all cases. This is a possible explanation why the prevalence of grade II-III toxicity and moist desquamation in our study was rather similar to the data derived from the IMRT series than from the early 3D-CRT series. Our results are in accordance with findings of Pastore et al. [15], who evaluated 140 consecutive breast cancer patients undergoing conventional three- dimensional conformal radiotherapy (3D-CRT) after breast conserving surgery in a prospective study assessing radiation induced skin toxicity. In their study additional field segments were used and the maximum dose in the treatment volume was limited to a level of < 107%. The most relevant factor regarding the occurance of skin toxicity in their study was the volume that received a dose of > 30 Gy. In our study, V80% (V40Gy) was the only parameter that was confirmed to have a significant impact on moist desquamation on multivariate analyses. V107% and V105% had no significant influence on multivariate analyses. Several studies reported that moist desquamation is experienced in the course of treatment after cumulative radiation doses of the skin in excess of 30–40 of 50 Gy [10, 18, 19] This suggests that moist desquamation does not occur until a certain threshold dose is reached. Once the threshold is reached the risk of developing moist desquamation depends on the volume of the skin exceeding this dose, measurable as the V40Gy or V30Gy respectively.

Previous studies reported mostly consistently that the breast volume is an important impact factor on the occurrence of acute skin toxicity [11, 12, 14]. Nevertheless, no significant coherence in regard to breast size was found during univariate analysis in the study of Pastore et al. [15]. As shown in our study there is a moderate correlation between breast size and the V80% skin. Nevertheless V80% was confirmed to have a significant impact on moist desquamation even if the breast volume was included in multivariate analysis.

It is critical to suggest constraints for the skin volume in order to reduce skin toxicity, since on the one hand estimation of the exact dose distribution within the skin is depended on the dose calculation algorithm and on the other hand dose coverage in the target volume must have the highest priority. However the results of Pastore et al. and our study reveal that the area receiving higher doses (> 30–40 Gy) in the skin should be as low as reasonable achievable.

A promising approach to reduce side effects is a skin sparing IMRT. Joseph et al. [20] achieved a reduction of moist desquamation from 33 to 11% by applying skinsparing helical tomotherapy in 177 patients in a phase III randomized controlled trial. Saibishkumar et al. [21] evaluated the feasibility of skin-sparing by configuring it as an organ-at-risk (OAR). Skin was contoured as a 4- to 5-mm strip extending from the patient outline of the breast planning target volume (PTV) in 14 patients. The mean skin dose and volume of skin receiving 50 Gy were significantly less with the skin-sparing plan compared with non–skin-sparing plan (42.3 Gy vs. 47.7 Gy and 12.2% vs. 57.8%). However no correlation to the clinical outcome was performed.

Boost irradiation in our study had different locations in the breast and had different total doses. As the location of moist desquamation was not assessed in this retrospective study, only the dose to the whole breast was analyzed during dose evaluation. Even though no significant impact of boost irradiation was found on the occurrence of skin toxicity in the current and previous studies, neglecting the boost during dose analysis is a potential limitation of our study [12]. Furthermore different fractionation schemes may lead to a different effect of dose distribution and the occurrence of skin toxicity. Even though we calculated the EQD2, our results may not be valid for hypofractionated whole breast irradiation.

## Conclusion

Modern tangential multi-field 3D-CRT allows a homogeneous dose distribution within the CTV and limits the hot spots within in the target volume and skin. The incidence of skin toxicities in this study was comparable to data from studies using IMRT for the adjuvant radiotherapy of the breast. To reduce skin toxicity, hot spots in the CTV (V > 107%) and skin (V > 105%) as well as the volume that receives more than 40Gy in the skin should be kept as low as reasonably achievable.

## Abbreviations

CTV: Clinical target volume
PTV: Planning taget volume
IMRT: Intensity modulated radiation therapy
3D-CRT: Three-dimensional conformal radiotherapy
kVCT: Kilo-voltage computed tomography
RTOG: Radiation Therapy Oncology Group
Dmax: Maximum dose
Dmean: Mean dose
CTCAE: Common Terminology Criteria for Adverse Events
Vxx: Absolute volume (cm3) receiving ≥110% (V110%), 107% (V107%) and 105% (V105%) of the prescribed dose
OAR: Organ-at-risk
